# EOGNET: A Novel Deep Learning Model for Sleep Stage Classification Based on Single-Channel EOG Signal

**DOI:** 10.3389/fnins.2021.573194

**Published:** 2021-07-12

**Authors:** Jiahao Fan, Chenglu Sun, Meng Long, Chen Chen, Wei Chen

**Affiliations:** ^1^Center for Intelligent Medical Electronics, School of Information Science and Technology, Fudan University, Shanghai, China; ^2^Human Phenome Institute, Fudan University, Shanghai, China

**Keywords:** deep learning, feature extraction, sleep stage classification, electrooculography, hierarchical neural network

## Abstract

In recent years, automatic sleep staging methods have achieved competitive performance using electroencephalography (EEG) signals. However, the acquisition of EEG signals is cumbersome and inconvenient. Therefore, we propose a novel sleep staging approach using electrooculogram (EOG) signals, which are more convenient to acquire than the EEG. A two-scale convolutional neural network first extracts epoch-wise temporary-equivalent features from raw EOG signals. A recurrent neural network then captures the long-term sequential information. The proposed method was validated on 101 full-night sleep data from two open-access databases, the montreal archive of sleep studies and Sleep-EDF, achieving an overall accuracy of 81.2 and 76.3%, respectively. The results are comparable to those models trained with EEG signals. In addition, comparisons with six state-of-the-art methods further demonstrate the effectiveness of the proposed approach. Overall, this study provides a new avenue for sleep monitoring.

## Introduction

Sleep-stage classification plays an essential role in sleep quality assessment and sleep disorder diagnosis. According to the american academy of sleep medicine (AASM), sleep stages can be categorized into five stages: wake, N1, N2, N3, and rapid-eye-movement (REM) (Iber, 2007). Sleep technicians generally use polysomnography (PSG), comprising a set of physiological signals, such as electroencephalography (EEG), electrooculography (EOG), and electromyography (EMG), to classify sleep stages. However, this process is tedious and time-consuming.

Numerous machine learning-based methods for automatic sleep staging have been proposed. Most studies use EEG signals as the primary modality (Längkvist et al., 2012; Sharma et al., 2017; Supratak et al., 2017; Chambon et al., 2018; Dong et al., 2018). Cardiorespiratory or movement signals are also explored to score sleep stages (Domingues et al., 2014; Willemen et al., 2014; Fonseca et al., 2017; Wei et al., 2018; Zhang et al., 2018). Generally, EEG-based algorithms can achieve high accuracy (Längkvist et al., 2012; Supratak et al., 2017; Chambon et al., 2018; Dong et al., 2018). However, the acquisition of EEG signals is relatively complex and may disturb natural sleep or alter sleep patterns. or alter sleep patterns.

In contrast, the Cardiorespiratory and movement signals are convenient to acquire (Chen et al., 2019). However, these methods are still in the exploratory stage, and their performance is unacceptable to clinicians. Therefore, a user-friendly approach with high accuracy for sleep-stage classification is required.

**FIGURE 1:**
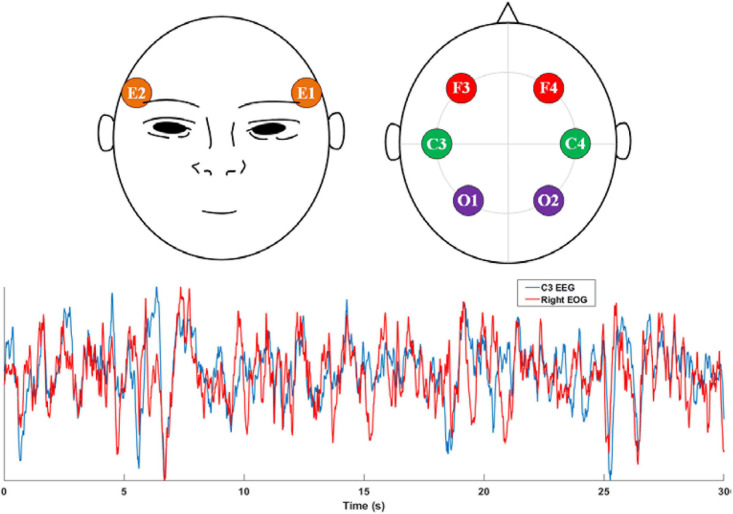
Top: the electrode placement positions of EOG and EEG signals recommended by AASM. E1 and E2 electrodes are used to acquire the left and right EOG, respectively. F3, F4, C3, C4, O1, and O2 are the most-used EEG electrodes in sleep monitoring; Bottom: comparison between C3 EEG signal and the Right EOG (E2) signal.

Considering the trade-off between feasibility and accuracy, we found that the EOG is a potential modality for sleep staging. First, EOG recordings can reflect eye activity, which is a crucial indicator for recognizing non-REM and REM stages. Second, EOG signals are typically contaminated with EEG signals. As shown in Figure 1, a high similarity between the EEG C3 channel and the EOG E2 channel was observed. Finally, EOG signals are generally convenient to acquire due to the ease of electrode placement. To exploit the feasibility of using standalone EOG signals to predict sleep stages, we designed a two-stage neural network to capture both temporary-equivalent features and sequential patterns from raw EOG signals. We used a two-scale convolutional neural network (CNN) to learn high-level features in the first stage. A recurrent neural network (RNN) captures the sequential information, especially the transition rules within sleep epochs, in the second stage. Compared with existing works (Sun et al., 2019b,a), the proposed method can achieve promising sleep staging performance from single-channel EOG signals.

The contributions of this paper are as follows.

(1) A novel sequential hierarchical neural network for sleep-stage classification using single-channel EOG signals is proposed to balance the complexity of data acquisition and accuracy of data analysis.
(2) To achieve competitiveness in sleep staging classification, the characteristic and temporal information within successive sleep epochs of EOG signal are explored.
(3) The proposed method is validated by comparing it with six existing state-of-the-art approaches.

The main context of this study is as follows. Section “Materials and Methods” details the methodology. The experimental process is described in section “Experiments.” The results are presented in section “Results,” and section “Discussion” discusses the experimental results and model analysis. The last section summarizes this study.

## Materials and Methods

**FIGURE 2:**
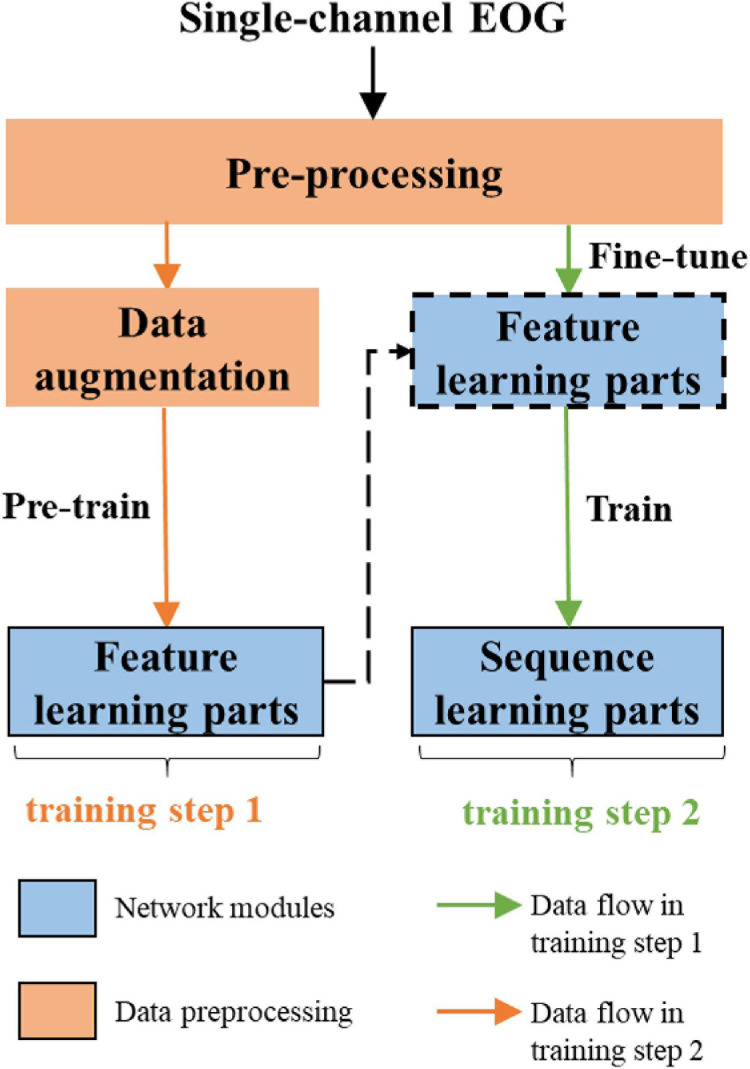
The overall workflow of the proposed method.

The overall workflow of the proposed approach is shown in Figure 2. The network consists of two parts, feature learning and sequence learning parts. The network is optimized with single-channel EOG with two-step training. In the first training step, the feature learning parts of the network are pre-trained. In the second training step, the learnable network weights of both feature learning, and sequence learning parts are optimized with a different learning rate. The detailed description of the proposed method is as follows:

### Feature Learning Parts

**FIGURE 3:**
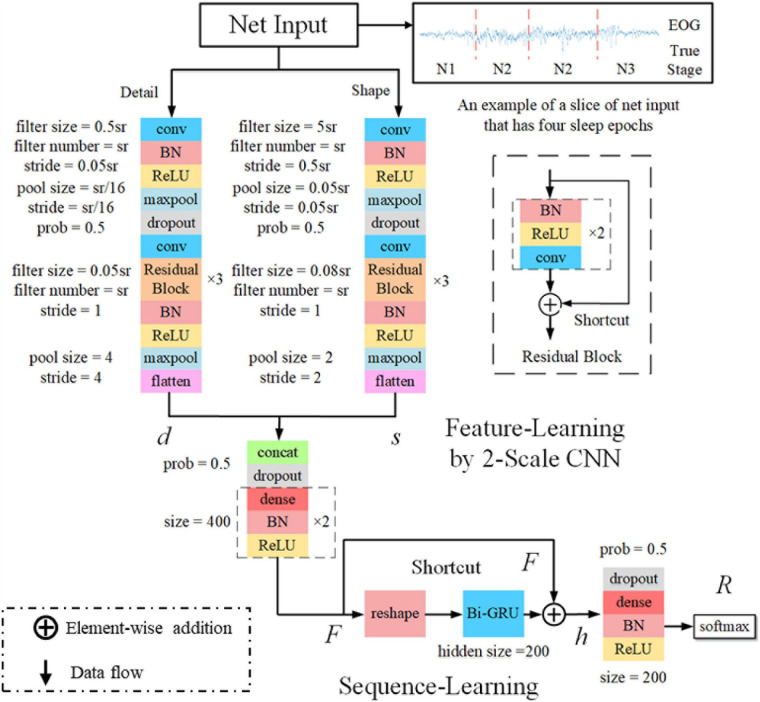
The architecture of the proposed network. sr, sampling rate of the input signals; conv, convolutional blocks; BN, batch normalization; ReLu, the rectified linear unit; *d* and *s*, features learned by feature learning parts; *F*, integrated features; *h*, the output of Bi-GRU; *R*, the predicted probabilities by the network.

The classification performance of the existing machine learning method primarily depends on feature engineering. However, extracting task-relevant features is challenging and complicated. In contrast, CNN-based approaches have powerful abilities of feature learning and have achieved high accuracy in many studies (Tsinalis et al., 2016; Supratak et al., 2017; Andreotti et al., 2018; Chambon et al., 2018; Dong et al., 2018). Inspired by previous studies, we designed a two-scale CNN to extract features with different temporal sizes and frequency resolutions from the EOG signal. As shown in Figure 3. the network consists of two CNN modules that capture features from different perspectives. CNN with small filter sizes and strides is in charge of extracting detailed features and high-frequency information. On the contrary, CNN with larger sizes and strides is to capture low-frequency information, such as sleep waves. Consequently, features extracted from two CNNs were concatenated, yielding a comprehensive feature to be further processed by the sequence learning parts.

### Sequence Learning Parts

Several stage-switch criteria and temporal relations (Hungs, 2012) exist in sleep recordings. Proficient sleep experts typically utilize these relations and criteria to score the present sleep epoch according to its adjacent epochs. Therefore, sequential modeling is of importance to capture inner-epoch patterns. In our study, the bidirectional RNN, which is realized by Gated Recurrent Unit (GRU) cells, is used to explore the sequential structure lying in EOG signals. As shown in Figure 3. The Bi-GRU cells receive features learned by the previous parts of the network, yielding sequential information. Shortcut connection is used to reserve residual information and avoid overfitting. This process can be presented as follows:

(1) h=B⁢i⁢G⁢R⁢U⁢(F)

(2) R=s⁢o⁢f⁢t⁢m⁢a⁢x⁢(h+F)

where, *BiGRU* and *softmax* represent Bi-GRU and softmax layer, respectively. *F*, *h*, and *R* denote features learned by feature learning, Bi-GRU, and the final predicted classification probabilities, respectively.

### Data Augmentation

**FIGURE 4:**
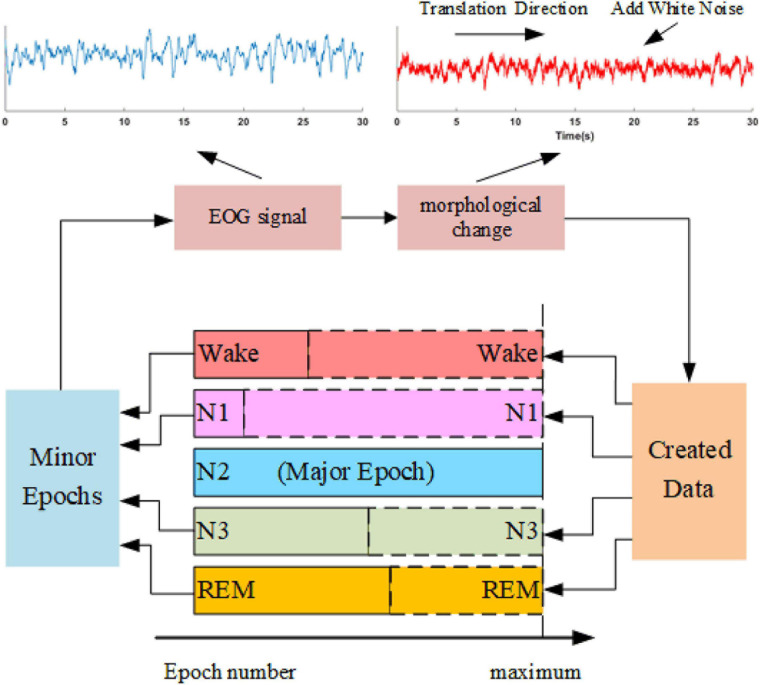
The oversampling approach is based on data augmentation. An example of the morphological operation is shown in the upper part. The blue waveform is the original EOG signal, and the red waveform suffers one translation with a length of 15 s and adds white noise with an SNR of 10 dB.

Sleep datasets suffer from class imbalance problems (CIPs). Several studies have attempted to address CIPs by oversampling minority class samples (Supratak et al., 2017; Fan et al., 2020). Such approaches can alleviate weight bias in the networks but fail to produce new patterns to improve the performance of trained models further. In this study, we propose a data augmentation approach, as shown in Figure 4. The method synthesizes sleep epochs for minor sleep stages to ensure that all sleep stages are equal in sample number in the training set. The samples are generated by morphological transformation, including translation operation and noise addition. To be specific, for each 30-s signal, a transition spanning 5 to 25 s is conducted along the time axis. Gaussian noise with a signal-to-noise ratio between 8 and 12 dB is added to the signals. The process is shown in Figure 4.

### Two-Step Training

**FIGURE 5:**
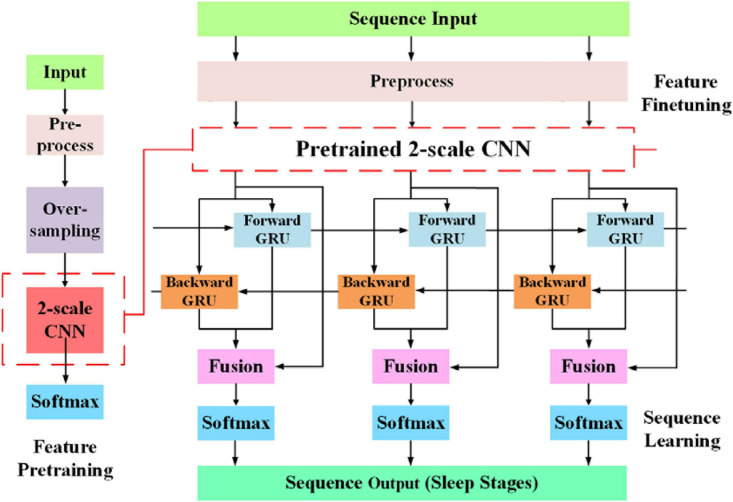
The entire training process, which can consider both oversampling and sequence information learning.

As shown in Figure 5, the network is optimized by two-step training. In the first step, to alleviate the CIPs in sleep training data, we use the proposed data augmentation method to ensure samples of all sleep stages equal in number. Then, with a softmax layer stacked on the top of two CNN layer, the feature learning parts of the network is pre-trained. By minimizing the cross-entropy loss between true labels and predicted scores, the weights of feature learning parts of the model are optimized. In the second step, we train the whole network end-to-end using sequence input, which keeps the original order of epochs in the sleep records unchanged. Due to the feature learning parts of the network is already trained, we used a lower learning rate to adjust the learned weight.

## Experiments

### Data

**FIGURE 6:**
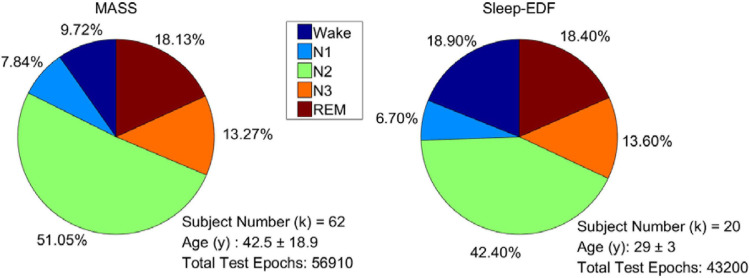
The data distribution of the MASS and Sleep-EDF databases.

For evaluating the performance of the proposed model, 101 full-night sleep data from the montreal archive of sleep studies (MASS) database (O’Reilly et al., 2014) and Sleep-EDF database (Goldberger et al., 2000) were used. The two databases are both open access and public. The MASS database, collected by the Sacred Heart Hospital of Montreal and Montreal University, has five subsets (SS1–SS5). We used all the 62 full-night PSG data collected from healthy people in the SS3 subset as it was labeled according to AASM. The recordings consist of 21 EEG channels, 2 EOG channels, and 3 chin EMG channels. The Sleep-EDF database contains two subsets: the Sleep Cassette (SC) and the Sleep Telemetry (ST). All 39 full-night recordings from 20 healthy people in the SC subset (all subjects have two night’s recordings except one subject) were used in the experiments. The subset contains two EEG channels, one horizontal EOG, and one chin EMG. Right EOG in MASS and horizontal EOG in Sleep-EDF were selected to train the proposed network. Signals were downsampled to 128 Hz to reduce computational complexity. Each signal was filtered by a band-pass filter of 0.3 to 35 Hz. The data distribution of each dataset is presented in Figure 6.

### Training Parameters

The filter sizes for the top CNN layers on two branches are set as 0.5 and 5 times the sampling rate, respectively. These values are set mainly based on the frequency features of sleep waves. The hyperparameters of the network are set as recommended in previous studies. The sequential information of the learned features by the pre-trained model was captured using a two-layer Bi-GRU. The Bi-GRU structure processed the input with a sequence length of 15 sleep epochs (signals spanning 450 s), which empirically indicated a higher accuracy. The weights of the pre-trained feature learning parts were fine-tuned with a learning rate of 10^−6^, while the learning rate for the sequence learning parts was set to 10^−4^. In addition, we employed a heuristic gradient clipping approach to avoid the gradient explosion problem. We used a couple of improved techniques, including ℓ−2 regularization, dropout technique (Srivastava et al., 2014), and focal loss (Lin et al., 2018), to improve the robustness and avoid overfitting of the trained models. The network is optimized using Adam optimizer (Kingma and Ba, 2017). The hyperparameters used in our experiments are summarized in Table 1.

**TABLE 1:** Network hyperparameters.

Hyperparameters	Value
Feature pretraining	

Batch size	100
Input dimension	(100, 3840, 1, 1)
ℓ−2 regularization term	10^–3^
Learning rate	10^–4^
*β*_1_ and *β*_2_	0.9, 0.999

Feature fine-tuning and sequence learning	

Batch size	10
Input dimension	(150, 3840, 1, 1)
Hidden size of Bi-GRU	200
*α* and *γ* in the focal loss	0.25 and 2
Learning rates	10^–6^, 10^–4^

*Batch, the set of examples used in one iteration; ℓ−2 regularization, regularization that penalizes weights in proportion to the sum of the squares of the weights; *β*_1_ and *β*_2_, coefficients of Adam optimizer to adjust the learning rate.

### Experimental Setting

The experiments were conducted under two protocols: the 5-class-task protocol and the 4-class-task protocol. In the first protocol, sleep stages were categorized into five classes, which was consistent with the staging criteria of AASM. In the second protocol, sleep stages were reformulated as stage Wake, Light, Deep, and REM, in which class Deep contains stage N1 and N2 defined in AASM. This criterion is practical for clinical applications as N1 is exceptionally scarce in sleep recordings. In both protocols, we used a leave-one-subject-out (LOSO) validation to evaluate the performance of the trained model. The overall accuracy (Acc.), F1-score (F1), Cohen’s kappa coefficient (κ), as well as precision, and recall are reported in this study.

## Results

### Overall Performance

The overall performance of the proposed approach is presented in Table 2. The results show that the model can attain a promising classification accuracy with 81.2% and 76.3% on MASS and Sleep-EDF for the 5-class task, and 85% and 82.1% on MASS and Sleep-EDF for the 4-class task, respectively. Besides, the high F1 score and κ indicate that the model can also accurately recognize minority classes. Overall, the results suggest that the proposed method using single-channel EOG performed as well as the method using EEG or other multi-modality inputs, demonstrating standalone EOG signals can be used as the primary modality to train automatic sleep staging models.

**TABLE 2:** Overall and class-wise performance.

5-class task (%)						
	**MASS**			**Sleep-EDF**		
	**Prec.**	**Rel.**	**F1**	**Prec.**	**Rel.**	**F1**

Wake	72.3	86.7	78.8	76.2	87.9	81.6
N1	54.0	40.7	46.4	33.9	36.0	34.9
N2	85.6	89.5	87.5	80.1	83.2	81.6
N3	80.4	64.4	71.5	73.8	71.2	72.5
REM	83.5	84.9	84.2	88.0	66.4	75.7

Overall	**Acc.**	**F1**	κ	**Acc.**	**F1**	κ
	
	81.2	73.7	71.8	76.3	69.3	67.2

**4-class task (%)**						

	**MASS**			**Sleep-EDF**		
	**Prec.**	**Rel.**	**F1**	**Prec.**	**Rel.**	**F1**

Wake	74.7	84.8	79.4	73.5	93.6	82.4
Light	87.4	90.1	88.7	84.2	84.6	84.4
Deep	80.0	66.4	72.5	83.7	69.9	76.2
REM	86.1	81.8	83.9	88.2	72.5	79.6

Overall	**Acc.**	**F1**	κ	**Acc.**	**F1**	κ
	
	85.0	81.1	74.3	82.1	80.6	73.3

**FIGURE 7:**
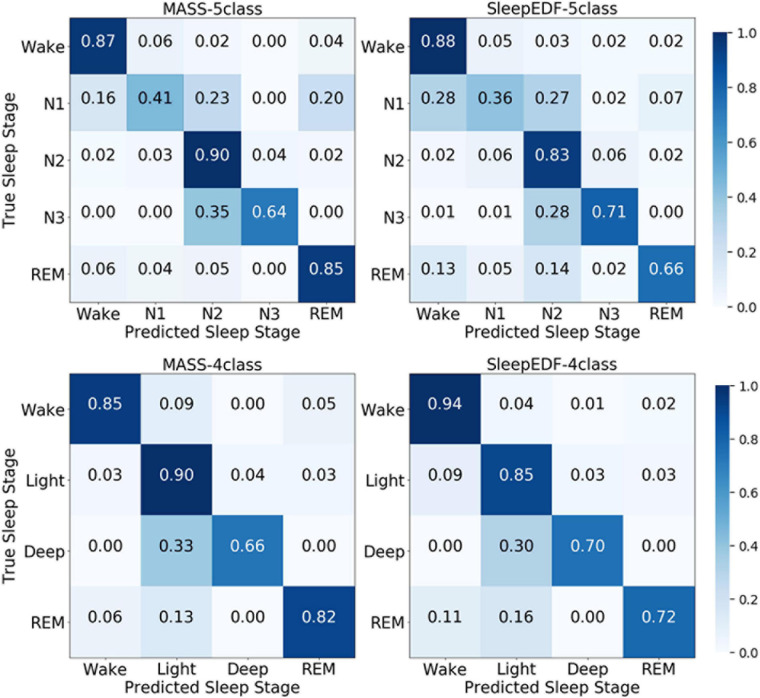
The normalized confusion matrices in the first and second row are obtained via the 5-class task and 4-class task, respectively. The first and second columns are obtained from the MASS and Sleep-EDF databases, respectively.

According to the confusion matrix in Figure 7, the class-wise accuracy of stage wake, N2, N3, and REM is relatively high both on the MASS and Sleep-EDF dataset for the 5-class task. In contrast, the accuracy for recognizing stage N1 is inferior to that of other stages. The results are consistent with methods using EEG, as N1 is excessively rare in sleep recordings. For the 4-class task, the class-wise accuracy for stage wake, light, deep, and REM range from 66 to 85%, which can meet the requirements for practical applications.

**FIGURE 8:**
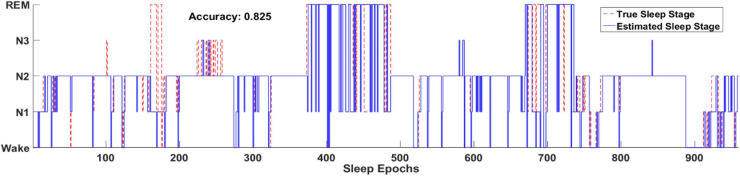
Two full-night hypnograms of one subject. The solid blue line and red dashed line denote the hypnograms depicted by the proposed model and a physician, respectively.

Figure 8 illustrates one example of the output hypnogram and its ground truth during about 8 h. It can be observed that the hypnogram predicted by the model aligns well with the ground truth. Most misclassified epochs can be found during stage transition, indicating the difficulty of recognizing the transitioning epochs. Nevertheless, most stage transitioning pairs could be accurately predicted by the proposed methods, such as N1-N2, N2-wake, and REM-N2,.etc.

### Comparison With Related Methods

We compared the proposed model with six state-of-the-art sleep staging approaches as described below:

#### Method 1

Willemen et al. (2014) combined a support vector machine (SVM) with an RBF kernel to conduct a 4-task classification. Features were extracted from the cardiorespiratory and movement signals. The authors selected features based on the minimum redundancy maximum relevance feature selection method.

#### Method 2

Dong et al. (2018) used a rectifier neural network (ReNN) to extract high-level features from the knowledge-based features, which were sequentially used for sequential learning. The stochastic gradient descent (SGD) approach and cross-entropy loss function were used to train the model without regularization.

#### Method 3

Längkvist et al. (2012) extracted 28 features from multimodal sleep data to train a deep belief network (DBN). A 2-layer DBN combined with a softmax classifier was used. Both layers were pre-trained for 300 epochs, and the top layer was fine-tuned for 50 epochs using modified z-score normalization. Finally, the output from the DBN was used as the input to a hidden Markov model (HMM) for final prediction.

#### Method 4

Tsinalis et al. (2016) extracted 557 EEG features to train a stacked sparse autoencoder (SSAE). The limited-memory Broyden–Fletcher–Goldfarb–Shanno approach was used for optimization. This method used regularization to prevent overfitting and utilizes the sparsity weight to control the scale of the sparsity penalty term.

#### Method 5

Chambon et al. (2018) proposed a CNN-based network for sleep staging by exploring temporal information among sleep epochs. The network was trained with batched data, in which each class is equal in number. We re-implemented the network and trained it using EOG and EEG signals separately for comparison.

#### Method 6

Supratak et al. (2017) proposed a sleep staging network with representation-learning and sequence residual learning parts. The former part extracted time-invariant features using two CNN components. The latter part used a two-layer bidirectional Long Short-Term Memory network (LSTM) (Chung et al., 2014; Greff et al., 2017) for sequential modeling. The authors attempted to address CIPs by repeating samples of minority classes. We re-implemented the network and trained it using EOG and EEG separately for comparison.

Compared to the proposed method, method 1, method 2, method 3, and method 4 are based on handcrafted features, which highly depend on the expertise knowledge. These features are partly explainable. However, a suboptimal feature set may lead to information loss. Thus the performance of methods using handcrafted features often inferior to those using feature-learning-based methods. In contrast, method 5 uses an end-to-end CNN model to learn compact and discriminative features from raw EEG signals. The sequential information is derived from five consecutive epochs. However, the model does not take long-term sequential information into account. Method 6 shares a similar concept with the proposed method using CNN modules and LSTM to extract time-invariant features and sequential information, respectively. However, the proposed method uses residual blocks on both CNN branches to reserve the learning capacity for shallow parts of the network. In addition, we proposed a data augmentation method to address sleep CIPs.

For fair comparisons, all the above models were trained using the MASS database on the same hardware platform. The hyperparameters were kept consistent with their original settings. LOSO validation was used for performance evaluation.

Table 3 summarizes the results of the comparisons. First, feature learning-based methods outperformed all the methods using knowledge-based handcraft features no matter the models were trained with EEG or EOG. This demonstrates the advantages of a neural network in extracting time-invariant features from raw physiological signals. Second, models trained with EEG have superior performance than those trained with EOG. This is consistent with our previous analysis. For EOG, the components from EEG play the most crucial role in recognizing sleep stages. Finally, among all the models trained with EOG, the proposed method has achieved the highest Acc, F1, and, κ corresponding to 81.2, 73.7, and 71.8%, respectively, which is comparable to its counterpart trained with EEG. This indicated that EOG is a potential modality to be used for sleep staging. The obtained accuracy (81.2%) can meet the requirements for community health care, home-based sleep monitoring, or even clinical applications.

**TABLE 3:** Results comparison of different methods using different features, classifiers, and signals.

Methods	Feature type	Method	Input	Overall results (%)			Class-wise F1-score (%)				
				Acc.	F1	κ	W	N1	N2	N3	REM
Willemen et al. (2014)	Handcraft	SVM	EOG	63.2	47.3	42.9	37.6	18.9	75.3	40.2	64.3
Dong et al. (2018)	Handcraft	ReNN + RNN	EOG	67.9	59.3	53.0	59.1	26.9	76.9	63.3	70.3
Längkvist et al. (2012)	Learned	DBN + HMM	EOG	72.4	66.7	62.2	72.7	38.3	79.4	72.7	70.5
Tsinalis et al. (2016)	Handcraft	SSAE	EEG	76.2	70.2	66.9	68.1	41.5	82.8	80.4	78.2
Chambon et al. (2018)	Learned	CNN	EEG	75.1	68.6	65.6	73.5	31.6	82.1	78.4	77.3
			EOG	69.2	60.9	54.8	61.6	29.4	77.6	64.8	71.3
Supratak et al. (2017)	Learned	CNN+RNN	EEG	83.4	77.9	75.3	82.1	55.3	88.1	77.7	86.1
			EOG	77.6	70.9	66.8	75.8	44.3	83.8	70.4	80.0
**Our model**	learned	CNN+RNN	EEG	83.1	76.4	74.5	82.2	50.8	88.5	74.3	86.1
			**EOG**	**81.2**	**73.7**	**71.8**	**78.8**	**46.4**	**87.5**	**71.5**	**84.2**

*The proposed method is marked in bold.*

### Model Analysis

To take a glimpse into how network components impact model performance, we conducted ablation studies. In particular, we analyzed and compared the performance of the proposed network in different settings: (1) dropping sequential learning parts; (2) without pre-training step; (3) replacing two-scale CNN with MobileNetV2 (Sandler et al., 2018) for representing learning; (4) replacing Bi-GRU with transformers (Vaswani et al., 2017) for sequential learning. Thereinto, MobileNetV2 is a well-known and efficient CNN-based feature extractor. It has achieved state-of-the-art performance on several tasks such as image recognition, object detection, and semantic segmentation. We tailored the original MobileNetV2 for sleep staging in our study. Transformer architecture has demonstrated impressive results for tasks with sequential modeling, such as audio recognition and natural language processing. We used it to capture sleep epoch transition rules in comparison with the proposed Bi-GRU module. The detailed introduction for these experimental settings can be found in Appendix 1.

**FIGURE 9:**
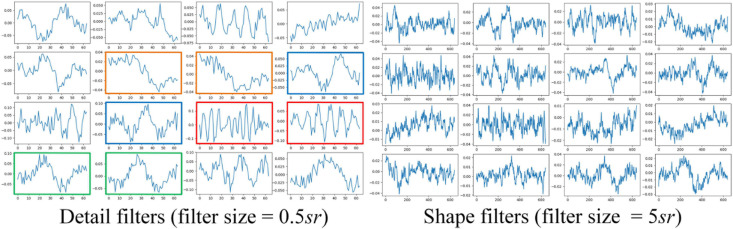
Examples of learned CNN filters in the feature learning parts of the network. Figures in green frames, learned filters that match characteristic waves during eye movements. Figures in red frames, filters that match spindle waves. Figures in blue frames, filters that match k-complex. Figures in yellow frames: filters that match slow waves.

The results of ablation studies are summarized in Table 4. Dropping sequential learning parts leads to a performance degeneration on both datasets. An accuracy decline from 81.2% to 76.0% on MASS and 76.3% to 72% on Sleep-EDF are observed, respectively. This demonstrates the importance of sequence learning. The temporal information captured by Bi-GRU contributes to a more accurate recognition of sleep stages. Training the network without the pre-training step also leads to an accuracy decrease of 1.2% and 0.6% on MASS and Sleep-EDF, respectively, indicating the pre-training step can facilitate the network to learn more generalized features from raw EOG signals. Replacing feature learning parts with MobileNetV2, one of the state-of-the-art feature extractors in many pattern recognition tasks, does not necessarily contribute to a further performance improvement. This indicates that the proposed two-scale CNN, which is designed based on the inherent characteristics of EOG signals, is capable of learning discriminative and compact features for sleep staging tasks. Features learned by the proposed two-scale CNN are visualized in Figure 9. CNNs with different receptive fields could capture complementary and task-relevant features from multiple perspectives. The CNN filters are optimized to match the time-invariant patterns, reflecting the characteristics of waves, such as k-complex and sleep spindles. This, to some extent, is equivalent to the sleep recording interpretation by human sleep technicians. Similarly, replacing Bi-GRU with transformer architecture also underperforms the proposed method. This suggests that Bi-GRU cells, at least in the context of sleep staging using single-channel EOG, are prior to the advanced network, i.e., transformers. In addition, we analyzed the feasibility and model complexity of the proposed network. As shown in Appendix Table A2 in Appendix 2, the proposed model is efficient and can meet the requirements for real-time evaluation on different hardware platforms, including mobile and wearable devices.

**TABLE 4:** Results of ablation studies.

MASS database Settings	Overall results (%)			Per-class F1-score (%)				
	Acc.	F1	κ	Wake	N1	N2	N3	REM
Dropping sequential learning	76.0	68.7	65.1	76.3	34.8	84.0	74.7	74.0
Without pre-training	80.0	72.3	69.8	78.0	43.4	86.4	71.6	82.4
Replacing two-scale CNN with MobileNetV2	78.2	70.8	67.5	74.9	43.4	85.2	68.3	82.1
Replacing Bi-GRU with transformer	73.5	69.2	62.5	76.6	39.6	83.6	79.0	66.9
**Proposed method**	**81.2**	**73.7**	**71.8**	**78.8**	**46.4**	**87.5**	**71.5**	**84.2**

**Sleep-EDF database**								

Dropping sequential learning	72.0	65.8	62.3	79.1	33.2	77.7	68.3	70.7
Without pre-training	75.7	68.0	66.7	78.1	27.9	80.7	74.6	78.7
Replacing two-scale CNN with MobileNetV2	75.1	68.5	66.0	79.2	30.9	79.0	73.2	80.3
Replacing Bi-GRU with transformer	70.3	65.7	58.1	72.6	36.2	81.2	74.5	63.8
**Proposed method**	**76.3**	**69.3**	**67.2**	**81.6**	**34.9**	**81.6**	**72.5**	**75.7**

*The proposed method is marked in bold.*

## Discussion

In this study, we proposed a novel automatic sleep staging network using single-channel EOG. The basis of using single-channel EOG signals for sleep staging is that EOG signals are typically multi-source, which mainly consist of frontal and ocular EEG activity, as well as eye muscle EMG activity. Many studies have exploited automatic sleep staging methods using single-channel EEG, achieving state-of-the-art performance. EOG signals, which contain rich information from EEG, are promisingly ideal alternatives as the primary modality to train sleep staging models. Moreover, EOG signals are generally convenient to acquire due to the ease of electrode placement. Thus EOG-based sleep staging approaches can promisingly provide a solution for long-term and home-based sleep monitoring.

To exploit the feasibility of using single-channel EOG to classify sleep stages. We develop a network to capture sleep patterns from raw EOG signals. The network consists of two parts, feature learning, and sequential learning parts. The weights of the network are optimized under a two-step training protocol. In the first step, the feature learning part is pre-trained to learn time-invariant features from raw EOG signals. In the second step, the sequential learning part of the network is trained to capture inner-epoch temporary information, and the weights of the feature learning part are fine-tuned. The experimental results on two publicly available databases indicate that the proposed methods can achieve comparable performance in comparison with methods using EEG. This indicates the feasibility of using single-channel EOG as the primary modality for sleep staging.

According to the analysis of six state-of-the-art sleep staging methods, using the same method, models trained with EEG can invariably outperform models trained with EOG signals. The rationale is that EEG patterns provide the dominant information for interpreting EEG recordings both for human sleep technicians and intelligence algorithms. With single-channel EOG, the proposed network has achieved the best results with an accuracy of 81.2% among all the methods. Consequently, the accuracy obtained from EOG-trained models can match that obtained from EEG-trained models with only marginal accuracy inferiority (1.9%).

The high accuracy is partly attributed to the topology of the networks, which is designed to match the characteristics of EOG signals, partly attributed to the two-step training protocol. To be specific, in the first step, the feature learning part of the network is pre-trained using class-balanced training sets, which are generated by our proposed data augmentation methods. The data augmentation method is inspired by the large-scale image recognition tasks in computer vision. Analog to images, morphological transformation on EOG signals can produce new samples with new patterns from origin signals, thus can improve the robustness of the trained models. The results of ablation studies suggest that all the components in the proposed network play essential roles in sleep staging. The performance of trained models drops when deprecating two-scale CNN, Bi-GRU, or two-step training protocols. Besides, we also tested several variants of the proposed network, including a network using MobileNetV2 to take the place of the proposed two-scale CNNs and a network using a transformer for sequential learning instead of proposed Bi-GRU cells. The results show that these variants can hardly further obtain performance gains but lead to performance degeneration.

Although the proposed method has achieved promising performance using single-channel EOG, several improvements are required in future works:

(1) In this study, all sleep recordings for model validation are from healthy subjects. Staging sleep recordings from patients with sleep disorders is considered to be more challenging. In future works, we plan to test the proposed methods on a larger population with different health conditions.
(2) For long-term sleep monitoring, the convenience of signal acquisition is a crucial factor, highly influencing usability and acceptance. Besides PSG and EOG signals can also be acquired by eye masks (Liang et al., 2015), glasses (Ishimaru et al., 2014), and even unobtrusive devices. Adjusting our method on EOG signals acquired from these devices is of great significance. Furthermore, cardiorespiratory or movement signals are also convenient for acquisition. Many studies have attempted to score sleep stages using the cardiorespiratory signals (Domingues et al., 2014; Willemen et al., 2014; Fonseca et al., 2017; Wei et al., 2018; Zhang et al., 2018). In future works, we will develop methods by combining such signals with EOG to improve the proposed method further.
(3) Sleep data from different cohorts generally suffer from data mismatch issues. Models trained using data from one database typically perform poorly on other databases. We plan to improve the generalization of the proposed method with several techniques such as transfer learning and meta-learning in our future work.

## Data Availability Statement

Publicly available datasets were analyzed in this study. This data can be found here: https://massdb.herokuapp.com/en/ and https://www.ahajournals.org/doi/full/10.1161/01.cir.101.23.e215.

## Author Contributions

CS, JF, CC, and WC: conceptualization and methodology. CS and JF: data curation. CC and WC: funding acquisition and supervision. CS: investigation and software. WC: project administration. CS and CC: writing-original draft. JF, CC, ML, and WC: writing-review and editing. All authors contributed to the article and approved the submitted version.

## Conflict of Interest

The authors declare that the research was conducted in the absence of any commercial or financial relationships that could be construed as a potential conflict of interest.

## Figures and Tables

**Appendix Figure A1:**
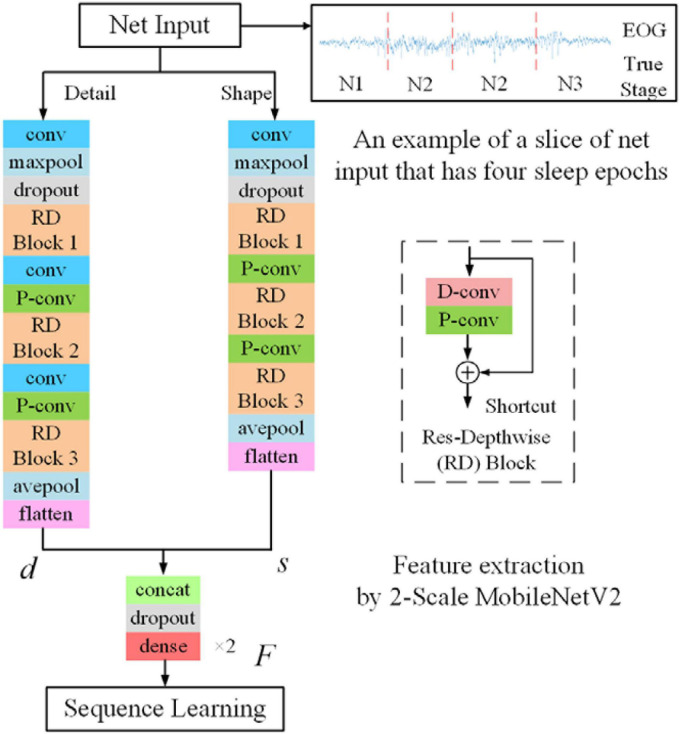
The feature-learning stage is based on the modified MobileNetV2. The RD block is shown on the right side. Each RD block consists of one D-conv layer and one P-conv layer with the shortcut connection.

## Appendix

### Appendix 1

Settings of Model Analysis. The descriptions of four settings used in ablation studies are as follows:

(1) Dropping sequential learning. In this scenario, Bi-GRU cells were removed from the network. A softmax layer is stacked on the top of feature learning parts to make predictions based on the learned features by two-scale CNNs.
(2) Without pre-training. Under this setting, training data without data augmentation is directly used for end-to-end network training.
(3) Replacing two-scale CNN with MobileNetV2: MobileNetV2 is a lightweight but efficient network for feature extraction, which can achieve state-of-the-art performance on many tasks, including image recognition, semantic segmentation, and object detection. The core of MobileNetV2 is depth-wise convolution blocks and shortcut connections. As presented in Appendix 3 and Appendix Table A1, we customized MobileNetV2 to suit the sleep staging application in our case and used it to take the place of the proposed two-scale CNN.
(4) Replacing Bi-GRU with a transformer: The transformer is a sequence to sequence network architecture, in which multi-head attention, shortcut connection, mask technique, and positional encoding were embedded. Recent studies have revealed the potential of transformers to be a powerful alternative for RNN. Accordingly, we attempted to use a transformer to play the role of sequential modeling instead of Bi-GRU cells.

### Appendix 2

Hardware realization feasibility of the method. The feasibility of the network is a crucial factor for practical application, especially for home-based monitoring applications. Therefore, we analyzed the complexity and computation cost of our trained models. In our analysis, time complexity (TC) and space complexity (SC) are indicated by floating-point operations (FLOPs) and the number of network parameters, corresponding to approximately O (3.95 × 10^7^) and O (1.67 × 10^6^), respectively. The required storage space for a single model is 12 Mb, which can be compressed to 2 Mb by quantization operation and pruning. Furthermore, we investigated the computation time of the trained models to predict on multiple platforms. As shown in Appendix Table A2, the model is very efficient and can meet the requirements for real-time evaluation, even on mobile and wearable devices.

**TABLE TA1:** Specifications of the MobileNetV2.

Detail feature extractor			

Layers	Size	Filter Number	Stride
Conv	0.5 *sr*	*sr*	0.05 *sr*
Max-pooling	*sr*/16	–	*sr*/16
RD Block 1	0.05 *sr*	*sr*	1
Conv	0.5 *sr*	2 *sr*	2
P-conv	1	2 *sr*	1
RD Block 2	0.05 *sr*	2 *sr*	1
Conv	0.05 *sr*	2 *sr*	2
P-conv	1	3 *sr*	1
RD Block 3	0.05 *sr*	3 *sr*	1
Avg-pooling	Full-size	–	Full-size

**Shape feature extractor**			

Conv	*sr*	*sr*	0.5 *sr*
Max-pooling	0.05 *sr*	–	0.05 *sr*
RD Block 1	0.05 *sr*	*sr*	1
P-conv	1	2 *sr*	1
RD Block 2	0.05 sr	2 *sr*	1
P-conv	1	3 *sr*	1
RD Block 3	0.05 *sr*	3 *sr*	1
Avg-pooling	Full-size	–	Full-size

**Feature concatenation**			

Dense	1000		
Dense	500		

** *sr* denotes the sampling rate of the preprocessed EOG signals.*

**TABLE TA2:** FLOPs per second of the current mainstream processors and their time consumptions for running the proposed model.

Processors	FLOPs per second (Theoretical peak)	Time consumption
NVIDIA GTX 1080 Ti (PC end)	11.3 T	3.5 μs
HUAWEI Kirin 970 (mobile end)	1.92 T	20.6 μs
Xilinx Cholesky (portable end)	20 G	2.0 ms
STM32F7 (wearable end)	20 M	2.0 s

### Appendix 3

Customized MobileNetV2. We develop a customized MobileNetV2 for sleep staging applications. Thereinto, a depth-wise separable convolution block is used to implement depth-wise and pointwise convolutions. Shortcut connections are used for learning the residual information. Finally, several Res-Depthwise (RD) blocks were stacked to serve as a feature extractor. The detailed architecture of the customized MobileNetV2 could be found in Appendix Figure A1 and Appendix Table A1.

## References

[B1] Andreotti F. Phan H. De Vos M. (2018). “Visualising convolutional neural network decisions in automatic sleep scoring,” in *Proc. Joint Workshop on Artificial Intelligence in Health (AIH)*, 2018 70–81.

[B2] Chambon S. Galtier M. N. Arnal P. J. Wainrib G. Gramfort A. (2018). A deep learning architecture for temporal sleep stage classification using multivariate and multimodal time series. *IEEE Transact. Neur. Syst. Rehabil. Eng.* 26 758–769. 10.1109/tnsre.2018.2813138 29641380

[B3] Chen C. Wang Z. Li W. Chen H. Mei Z. Yuan W. (2019). Novel flexible material-based unobtrusive and wearable body sensor networks for vital sign monitoring. *IEEE Sens. J.* 19 8502–8513. 10.1109/jsen.2018.2887107

[B4] Chung J. Gulcehre C. Cho K. Bengio Y. (2014). Empirical evaluation of gated recurrent neural networks on sequence modeling. *arXiv* [Preprint]. arXiv:1412.3555.

[B5] Domingues A. Paiva T. Sanches J. M. (2014). Hypnogram and sleep parameter computation from activity and cardiovascular data. *IEEE Trans. Biomed. Eng.* 61 1711–1719. 10.1109/tbme.2014.2301462 24845281

[B6] Dong H. Supratak A. Pan W. Wu C. Matthews P. M. Guo Y. (2018). Mixed neural network approach for temporal sleep stage classification. *IEEE Transact. Neur. Syst. Rehabil. Eng.* 26 324–333. 10.1109/tnsre.2017.2733220 28767373

[B7] Fan J. Sun C. Chen C. Jiang X. Liu X. Zhao X. (2020). EEG data augmentation: towards class imbalance problem in sleep staging tasks. *J. Neur. Eng.* 17:056017. 10.1088/1741-2552/abb5be 33055386

[B8] Fonseca P. Teuling den N. Long X. Aarts R. M. (2017). Cardiorespiratory sleep stage detection using conditional random fields. *IEEE J. Biomed. Health Inform.* 21 956–966. 10.1109/jbhi.2016.2550104 27076473

[B9] Goldberger A. L. Amaral L. A. Glass L. Hausdorff J. M. Ivanov P. C. Mark R. G. (2000). PhysioBank, PhysioToolkit, and PhysioNet: components of a new research resource for complex physiologic signals. *Circulation* 101 E215–E220. 10851218 10.1161/01.cir.101.23.e215

[B10] Greff K. Srivastava R. K. Koutník J. Steunebrink B. R. Schmidhuber J. (2017). LSTM: a search space odyssey. *IEEE Transact. Neur. Netw. Learn. Syst.* 28 2222–2232. 10.1109/tnnls.2016.2582924 27411231

[B11] Hungs M. (2012). Fundamentals of sleep medicine. *JAMA* 307 1320–1321.

[B12] Iber C. (2007). The AASM manual for the scoring of sleep and associated events: rules, terminology and technical specifications. *Am. Acad. Sleep Med.* 7:59.

[B13] Ishimaru S. Uema Y. Kunze K. Kise K. Tanaka K. Inami M. (2014). “Smarter eyewear-using commercial EOG glasses for activity recognition,” in *Proceedings of the 2014 ACM International Joint Conference on Pervasive and Ubiquitous Computing*, New York, NY. 10.1145/2638728.2638795

[B14] Kingma D. P. Ba J. (2017). Adam: a method for stochastic optimization. *arXiv* [Preprint]. arXiv:1412.6980.

[B15] Längkvist M. Karlsson L. Loutfi A. (2012). “Sleep stage classification using unsupervised feature learning,” in *Advances in Artificial Neural Systems*, London.

[B16] Liang S.-F. Kuo C.-E. Lee Y.-C. Lin W.-C. Liu Y.-C. Chen P.-Y. (2015). Development of an EOG-Based automatic sleep-monitoring eye mask. *IEEE Transact. Instrument. Measur.* 64 2977–2985. 10.1109/tim.2015.2433652

[B17] Lin T.-Y. Goyal P. Girshick R. He K. Dollár P. (2018). Focal loss for dense object detection. *arXiv* [Preprint]. arXiv:1708.02002. 10.1109/TPAMI.2018.2858826 30040631

[B18] O’Reilly C. Gosselin N. Carrier J. Nielsen T. (2014). Montreal archive of sleep Studies: an open-access resource for instrument benchmarking and exploratory research. *J. Sleep Res.* 23 628–635. 10.1111/jsr.12169 24909981

[B19] Sandler M. Howard A. Zhu M. Zhmoginov A. Chen L.-C. (2018). “MobileNetV2: inverted residuals and Linear bottlenecks,” in *Proceedings of the 2018 IEEE/CVF Conference on Computer Vision and Pattern Recognition*, Salt Lake City, UT. 10.1109/CVPR.2018.00474

[B20] Sharma R. Pachori R. B. Upadhyay A. (2017). Automatic sleep stages classification based on iterative filtering of electroencephalogram signals. *Neural Comput. Applic.* 28 2959–2978. 10.1007/s00521-017-2919-6

[B21] Srivastava N. Hinton G. Krizhevsky A. Sutskever H. Salakhutdinov R. (2014). Dropout: a simple way to prevent neural networks from overfitting. *J. Mach. Learn. Res.* 15 1929–1958.

[B22] Sun C. Chen C. Fan J. Li W. Zhang Y. Chen W. (2019a). A hierarchical sequential neural network with feature fusion for sleep staging based on EOG and RR signals. *J. Neural Eng.* 16:066020. 10.1088/1741-2552/ab39ca 31394522

[B23] Sun C. Chen C. Li W. Fan J. Chen W. (2019b). A hierarchical neural network for sleep stage classification based on comprehensive feature learning and multi-flow sequence learning. *IEEE J. Biomed. Health Inform.* 9:1. 10.1109/JBHI.2019.2937558 31478877

[B24] Supratak A. Dong H. Wu C. Guo Y. (2017). DeepSleepNet: a model for automatic sleep stage scoring based on raw single-channel EEG. *IEEE Transact. Neur. Syst. Rehabil. Eng.* 25 1998–2008. 10.1109/tnsre.2017.2721116 28678710

[B25] Tsinalis O. Matthews P. M. Guo Y. Zafeiriou S. (2016). Automatic sleep stage scoring with single-channel EEG using convolutional neural networks. *arXiv* [Preprint]. arXiv:1610.01683.

[B26] Vaswani A. Shazeer N. Parmar N. Uszkoreit J. Jones L. Gomez A. N. (2017). Attention is all you need. *arXiv* [Preprint]. arXiv:1706.03762v5 [cs.CL].

[B27] Wei R. Zhang X. Wang J. Dang X. (2018). The research of sleep staging based on single-lead electrocardiogram and deep neural network. *Biomed. Eng. Lett.* 8 87–93. 10.1007/s13534-017-0044-1 30603193 PMC6208558

[B28] Willemen T. Van Deun D. Verhaert V. Vandekerckhove M. Exadaktylos V. Verbraecken J. (2014). An evaluation of cardiorespiratory and movement features with respect to sleep-stage classification. *IEEE J. Biomed. Health Inform.* 18 661–669. 10.1109/jbhi.2013.2276083 24058031

[B29] Zhang X. Kou W. Chang E. I.-C. Gao H. Fan Y. Xu Y. (2018). Sleep stage classification based on multi-level feature learning and recurrent neural networks via wearable device. *Comput. Biol. Med.* 103 71–81. 10.1016/j.compbiomed.2018.10.010 30342269

